# Established method of chondroitin sulphate extraction from buffalo (*Bubalus bubalis*) cartilages and its identification by FTIR

**DOI:** 10.1007/s13197-018-3253-4

**Published:** 2018-07-18

**Authors:** G. Sundaresan, Robinson J. J. Abraham, V. Appa Rao, R. Narendra Babu, V. Govind, Mahantesh F. Meti

**Affiliations:** Department of Livestock Products Technology (Meat Science), Madras Veterinary College, Tamil Nadu Veterinary and Animal Sciences University, Chennai, 600 007 India

**Keywords:** Cartilages, Chondroitin sulphate, Buffalo, FTIR, SDS-PAGE

## Abstract

A study was conducted for extraction of chondroitin sulphate (CS) from buffalo tracheal, nasal and joint cartilages. CS was extracted from cartilages using 0.25% papain digestion, dialyzed, precipitated with 10% TCA and finally lyophilized to dry powder. Dimethylmethylene blue assay was performed to estimate the quantity of CS extracted. Identification of extracted CS was performed with SDS-PAGE and Fourier transforms infrared spectroscopy (FTIR). SDS-PAGE analysis of extracted CS revealed similar electrophoretic pattern to that of standard and the molecular weight ranged from 5 to 20 kDa. FTIR spectra of extracted CS revealed presence of characteristic peaks of –CONH vibration of amide group, coupling of C–O stretching vibration, S=O stretching vibrations and –C–O–S molecules confirms the CS moiety. It can be concluded that extraction method adopted could efficiently be utilized for the extraction of CS from buffalo by-products like tracheal, nasal and joint cartilages.

## Introduction

The buffalo meat industry is one of the largest established animal husbandry sector in India. According to FAO (2008) estimates, 107 million livestock were slaughtered annually in India leading to production of 6.3 million tonnes of meat. It leaves huge amount of by-products which accounts more than 10 million MT of edible and inedible by-products. The value of unprocessed by-products from buffalo in India was reported to be 30% (Chatterjee et al. 1991) which is quite high. Though no reliable data is available on the economic losses incurred to the nation, yet some estimates put it as Rs. 1000 crore/annum from slaughterhouses (Thota 1999). Effective utilization of inedible by-products generated during slaughter of buffaloes can lead to generation of value added products and minimizing the environmental pollution. Chondroitin sulphate is the most abundant naturally occurring proteoglycan in the extracellular matrix of the cartilage and connective tissues in animals (Manjusha and Saleena 2011). Buffalo tracheal, nasal and joint cartilages which are generally considered as inedible by-products could serve as a source of CS.

CS is an acidic polysaccharide comprised of repeated disaccharide units of *N*-acetylgalactosamine and glucuronic acid which is responsible for the resiliency and maintenance of structural integrity of the cartilage tissue, promotion and maintenance of cartilage structure and function. It also has been shown to elicit: anti-inflammatory effects, increase in type II collagen and proteoglycans, a reduction in bone resorption and a better anabolic/catabolic balance in chondrocyte. CS has the ability to bind receptor sites on synovial cell surfaces and thus induce production of hyaluronic acid, crucial to joint mobility and can bring significant pain relief and enhanced mobility in osteoarthritis (Nicola 2009; Somashekar et al. 2011).

CS has wide range of application in the pharmaceutical, cosmetic and food industries. In pharmaceutical application CS used as a chondroprotective (Dean et al. 1991), anti-arthrogenic, anti-anemic, anti-osteoporotic and also used for treatment of bladder cystitis, cancer, psoriasis and wound healing. CS along with glucosamines is used as an alternative medicine for treatment of osteoarthritis and it is currently recommended by the European league against rheumatism (EULAR) in the treatment of knee and hip arthritis (Jerosch 2011). Clinically chondroitin sulphate is used for treatment of osteoarthritis according to McAlinddon et al. (2000), wound healing Wang et al. (2006), CS aqueous solution containing 90–100 mg/ml for washing acute and chronic open wound to aid the natural healing Schiraldi et al. (2010).

Numerous research works have been carried out to extract CS from various slaughterhouse by-products such as bovine tracheal cartilage, chicken keel cartilage (Luo et al. 2002), bovine nasal cartilage (Nakano et al. 2000), porcine skin (Damle et al. 1979), pig laryngeal cartilage (Li and Xiong 2010), duck trachea (Vittayanont and Jaroenviriyapap 2014) and crocodile hyoid and sternal cartilage (Garnjanagoonchorn et al. 2007). Research literature pertaining to possibility of utilization of buffalo slaughterhouse by-products for extraction of CS is scant. Keeping in view of the above all facts a study has been planned for extraction and identification of CS from tracheal, nasal and joint cartilages of buffalo.

## Materials and methods

Buffalo tracheal, nasal and joint cartilages were collected hygienically from Corporation slaughterhouse, Chennai-12 and also the joint cartilage was collected from Brahramagiri slaughterhouse (Malabar Meat) located at Sulthan Bathery, Wayanad, Kerala and brought to the laboratory by maintaining cold chain. Ultra Refined Papain (EC. No. 232-73-4, activity 3000 USP/mg) from *Cercariya papaya* was obtained from M/S Himedia Pvt. Ltd. Mumbai. Absolute ethanol, acetic acid and trichloro acetic acid, sodium hydroxide pellets (pre analyzed) were obtained from M/S Fisher Scientific Pvt. Ltd., Chennai.

### Extraction of chondroitin sulphate

CS was extracted from buffalo tracheal, nasal and joint cartilages separately following procedure outlined by Vittayanont and Jaroenviriyapap (2014) with minor modifications in the concentration of papain used and time duration in extraction procedure. The cartilages collected from the slaughterhouse were defatted by soaking in 20% ethanol for 24 h and heat treated with 10 volumes of boiling water for 1 h and strained using strainer and retentate was hydrolyzed in equal volumes of 0.25% of papain solution at 65 °C by using thermostatic water bath (Lauda RE106) for 10 h. At the end of digestion, action of enzyme papain was inactivated by increasing the temperature to 100 °C for 10 min. The hydrolyzates were collected and centrifuged at 4 °C (Eppendorf, 5810R) for 15 min at 12,000 rpm. The supernatants obtained were collected and precipitated using 10% TCA solution at 4 °C for 12–18 h. Later the precipitates were removed by decanting followed by dialyzing using dialysis tube (HIMEDIA^®^ 3.63 ml/cm, molecular weight cut: 12 kDa) against 0.1 M acetic acid solution by changing the dialysis solution every 6 h for 24 h. The resultant material was collected and lyophilized by using lyophilizer (Christ Alpha 1-2 LDPlus).

### Quantitative analysis

The CS content in lyophilized powder was determined by DMMB assay as per the procedure outlined by Thomas-Coulson et al. (2014). Different concentrations (0, 1, 2, 3, 4, 5, 6 µg/ml) of standard chondroitin-4-sulphate (C4S) stock solutions were prepared along with DMMB dye was pipetted out in 96 well microplate and the volume was made up to 20 µl with distilled water. A volume of 20 µl from each extracted CS samples was pipetted out in subsequent wells containing 200 µl of DMMB dye in the microplate followed by shaking of the plate for 5 s using microplate shaker. The absorbance was recorded using UV–Vis spectrophotometer at 525 nm immediately and plotted against concentration on a graph and the CS content was determined by referring to the standard graph.

### Qualitative analysis

#### Qualitative analysis of CS extracted from buffalo tracheal, nasal and joint cartilages by FTIR

Qualitative determination of extracted CS (lyophilized) samples was performed by procedure described by Khan et al. (2013). Identification of CS component in the buffalo cartilages was done with FTIR (Bruker AVANCE III 500 MHz (AV 500), at Indian Institute Technology (IIT), Chennai) by comparing with standard spectrum of chondroitin sulphate with sample’s spectra.

#### Determination of molecular weight by SDS-PAGE

SDS–PAGE was carried out as per the procedure outlined by Vittayanont and Jaroenviriyapap (2014) with slight modification suggested by Manjusha and Saleena (2011) using 12% stacking gel and a 5% resolving gel with a constant current of 50 milliampere (mA). 20 µl of the sample was loaded in each well. The high molecular weight markers were used to estimate the molecular weight of the bands. After electrophoresis, the gels were stained with 0.1% (w/v) Coomassie Brilliant Blue R-250 in 50% (v/v) methanol and 6.8% (v/v) glacial acetic acid for overnight (12 h) and destained using 7.5% (v/v) of glacial acetic acid and 5% (v/v) methanol for about 9 h by changing the solution every 3 h of interval. The semi quantitative analysis of band intensity was done using the Gel Doc EZ Imager with the Image Lab 3.0 software (Bio-Rad Laboratories, Inc.).

## Results and discussion

The buffalo tracheal, nasal and joint cartilages are relatively low value by-products which were not often processed into high value products rather than transforming it into pet food or fertilizer in large scale processing industries. Whereas, in small scale processing these cartilages are mostly disposed off in land or by dumping in water bodies which ultimately leads to environmental pollution and sometimes spread diseases. Alternatively, cartilages can efficiently be utilized for production of high value naturally occurring proteoglycan CS, which is extensively distributed in the extracellular matrix of the cartilages.

### Quantitative analysis of extracted samples

**Fig. 1:**
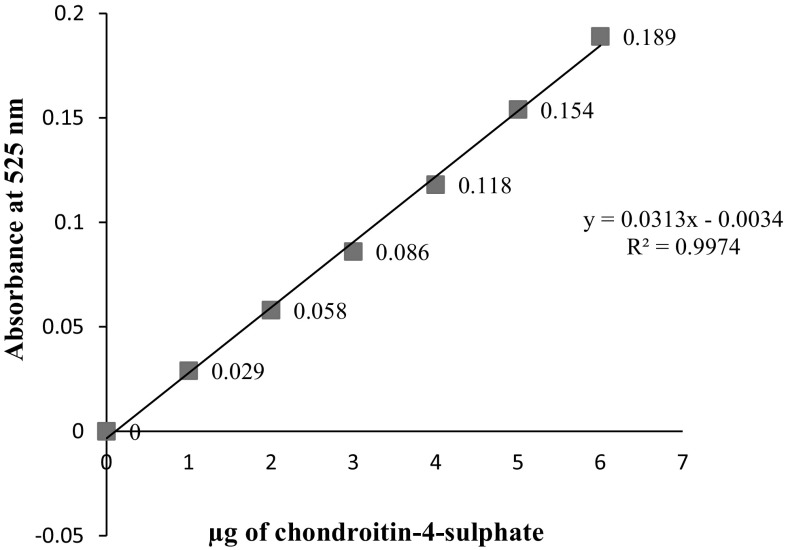
Calibration curve using chondroitin-4-sulfate sodium salt standard (bovine cartilage) by dimethylmethylene blue assay

Quantity of CS content in extracted samples from buffalo cartilages were determined by DMMB assay. Calibration curve was plotted between known concentration (1, 2, 3, 4, 5 and 6 µg/g) of standard CS and respective absorbance value (0.029, 0.058, 0.086, 0.118, 0.154, 0.189) at 535 nm showed linear relationship (Fig. 1). The CS concentration extracted from buffalo tracheal, nasal and joint cartilages were 62.05 ± 1.12, 60.47 ± 1.19 and 60.76 ± 0.38 mg/g respectively and ranged from 56 to 63.78 mg/g. These findings were in agreement with Khan et al. (2013) who had estimated per cent glycosaminoglycan (GAG) content samples from chicken keel cartilages at 525 nm was 70.77 ± 2.35% by the same method. Further Luo et al. (2002) also reported a GAG of 75.5 ± 4.2%. Bjornsson (1998) claimed that DMMB assay was the more popular method for quantitation of all sulfated GAGs in biological fluids without protease treatment. This was especially true in the research of articular cartilage, and synovial fluid. Garnjanagoonchorn et al. (2007) reported differences in the sulphated position of C4S and chondroitin-6-sulphate (C6S) affect the absorption at 525 nm. The yield of CS extracted from cartilage samples were determined by using calibration curve of C4S. Muller and Hanschke (1996) estimated proteoglycans in the cartilages by precipitation with 1,9 dimethylmethylene blue and concluded that consumption of dye directly related to amount of proteoglycans present in the extract.

This study revealed that the concentration of CS extracted from buffalo tracheal, nasal and joint cartilages were 62.05 ± 1.12, 60.47 ± 1.19 and 60.76 ± 0.38 mg/g respectively.

### Qualitative analysis

#### Qualitative analysis by FTIR

**Fig. 2:**
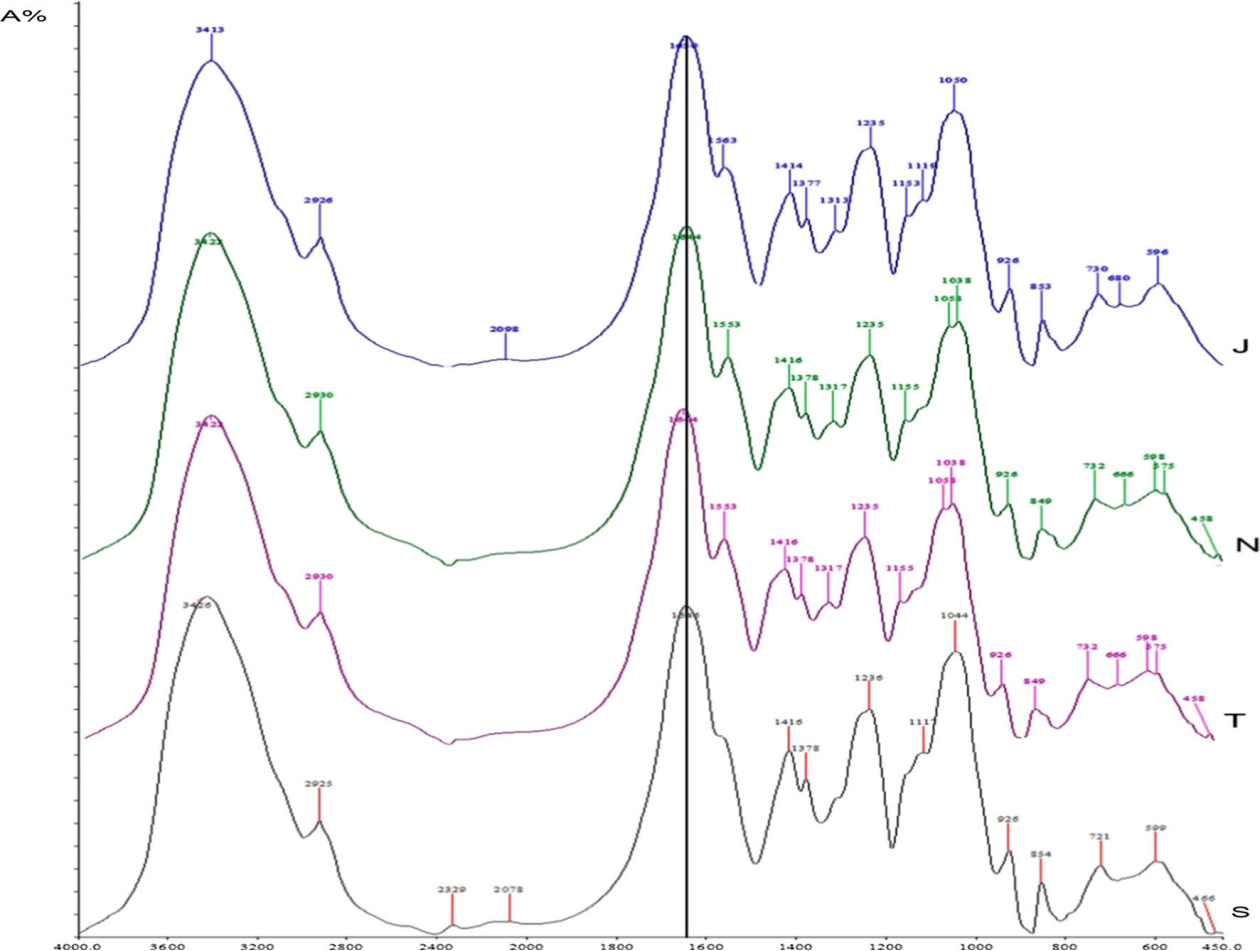
FTIR spectrum of S, standard bovine sodium chondroitin-4-sulphate; T, chondroitin sulphate from buffalo tracheal cartilage; N, chondroitin sulphate from buffalo nasal cartilage and J, chondroitin sulphate from buffalo joint cartilage. A%, absorbance

CS extracted from buffalo tracheal, nasal and joint cartilage were identified by FTIR spectroscopy at 400–4000 cm^−1^ and technique using potassium bromide pellet by comparing with standard CS. FTIR spectra of CS exhibited the characteristic peaks of –CONH vibration of amide group coupling of C–O stretching vibration, S=O stretching vibrations, –C–O–S, –COO; C–C, C–O–S and R–SO2–R; R–SO2–R as shown in Fig. 2. The characteristic peaks of –CONH was observed at 1646 cm^−1^ for standard CS and same was recorded in extracted samples of buffalo tracheal, nasal and joint cartilages are 1647, 1650, 1650 cm^−1^ respectively, and the results were in agreement with Khan et al. (2013) who had reported characteristic peaks of CS samples extracted from chicken keel cartilage –CONH vibration of amide group coupling of C–O stretching vibration at 1641 cm^−1^. Agustin et al. (2016) found that the FTIR spectrum of chondroitin isolated from shark cartilage had a broad and strong tape characteristic at the wavelength of 3000 cm^−1^ and strong absorption at the wave number of 1668.31, 1627.81, 1456.16, and 1415.65 cm^−1^ in the back part of cartilage, while the FTIR spectrum of side part showed that the chondroitin had a sharp and strong tape at the wavelength of 1672.17, 1627.81, 1454.23, and 1413.72 cm^−1^, respectively.

Sulityowati et al. (2015) also reported that FTIR spectrum of CS marketed in Indonesia had a peak of strong absorption at the wave number of 1637.63 and 1420.03 cm^−1^ indicating the presence of carboxyl groups, amine and sulphate. Strong peak recorded at 1627.81 and 1415.65 cm^−1^ in chondroitin isolated from the shark backbone was similar to the peaks of 1627 and 1413.72 cm^−1^ of side part of cartilage that indicates the existence of carboxyl group with amine and sulphate. Xiong et al. (2012) analyzed the CS from pig laryngeal cartilage by FTIR and reported that there was a strong absorption at 1650 and 1556 cm^−1^, corresponding to the stretching vibration of the cabonyl bond of the amide group and bending vibration of the N–H bond respectively, had shown the existence of acetamido group in chondroitin sulphate.

The characteristic peaks of C–O–S was observed at 854 cm^−1^ for standard CS and the same was recorded in extracted samples of buffalo tracheal, nasal and joint cartilages at 853, 853, 853 cm^−1^ respectively and results were in agreement with Xiong et al. (2012) who reported absorption band at 825.78 and 885.25 cm^−1^ attributable to the C–O–S axial and equatorial bending vibration are characteristics of 4-sulphate and 6-sulphate of d-galactosamine units and similar results reported by Garnjanagoonchorn et al. (2007) that the CS extract from shark fin cartilage, chicken keel cartilage, crocodile hyoid and sternal cartilage by FTIR spectroscopy potassium bromide pellet technique using C4S and C6S as a standard in which shark fin cartilage had shown a more distinct peak at 824 cm^−1^ while other cartilage extracts exhibited distinct peak at 857 cm^−1^, indicating that they consisted of different proportion of C4S and C6S.

The characteristic peaks of S=O was observed at 1236 cm^−1^ for standard CS and same was recorded in extracted samples of buffalo tracheal, nasal and joint cartilages at 1236, 1236, 1236 cm^−1^ respectively and results were in agreement with Khan et al. (2013) they were recorded characteristic S=O peaks of CS extracted from chicken keel cartilage as 1254 cm^−1^ with slight variation. The slight variation noticed in this study might be due to structural changes during the extraction procedure. Yin and Xia (2010) studied that the macromolecular concentration of bovine nasal cartilage proteoglycan by FTIR imaging and reported that infrared absorption peak areas between the 1072–1855 cm^−1^ can only be used as qualitative indicators of the molecular contents.

#### SDS-PAGE of extracted chondroitin sulphate samples

**Fig. 3:**
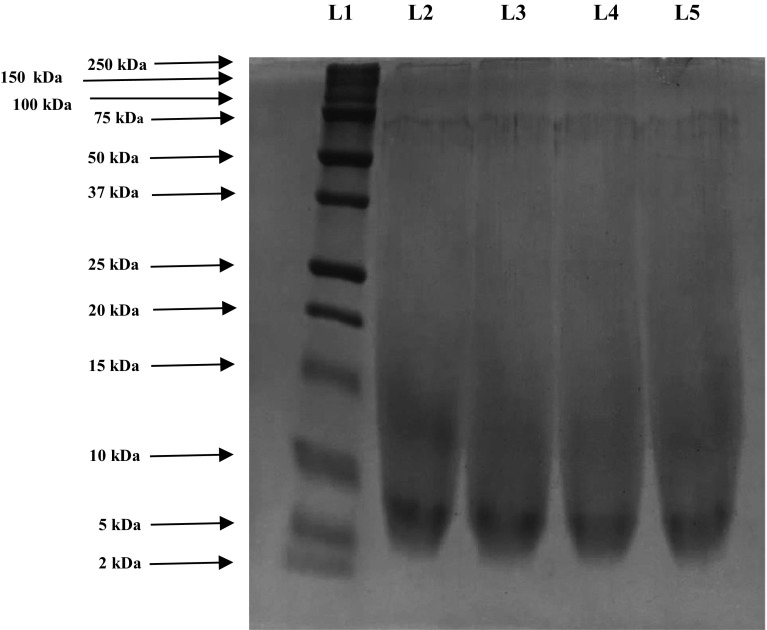
Molecular pattern of chondroitin sulphate extracted from buffalo cartilages on SDS, polyacrylamide gel electrophoresis (SDS-PAGE). Lane 1 (L1): protein marker, Lane 2 (L2): standard chondroitin sulphate. Lane 3 (L3): extracted chondroitin sulphate from tracheal cartilage. Lane 4 (L4): extracted chondroitin sulphate from nasal cartilage. Lane 5 (L5): extracted chondroitin sulphate from joint cartilage

Extracted samples (6 replicates) were identified by SDS-PAGE under denatured condition in 12% resolving gel. The SDS-PAGE patterns of CS extracted from buffalo tracheal, nasal and joint cartilages by papain was shown in the Fig. 3. The SDS-PAGE showed bands in lane 1 representing the protein marker were recorded in twelve bands with the molecular weight of 250, 100, 75, 50, 37, 20, 15, 10, 5 and 2 kDa in sequential order; whereas the standard CS from bovine and CS extracted from buffalo tracheal, nasal and joint cartilages in lane 2, lane 3, lane 4 and lane 5 depicted a band with molecular weight of between 5 to 10 kDa and also scattered band with the molecular weight ranged from 10 to 20 kDa. The pattern of CS movement on SDS-PAGE had shown no significant difference among CS samples extracted from buffalo tracheal, nasal and joint cartilages. The CS extracted from all three cartilages by papain had shown similar electrophoretic pattern as of standard CS (obtained from bovine tracheal cartilage).

Tomiosso et al. (2005) analysed the extracellular matrix GAG’s of ostrich articular cartilage by polyacrylamide gel electrophoresis followed by digestion with papain and showed that diffused bands on entire region contains only CS. In the present study electrophoretic movement of CS extracted from buffalo tracheal, nasal and joint cartilages by papain also shown diffused band pattern with single thick band at the molecular weight between 5 to 10 kDa. Zhang et al. (2009) characterized the GAG’s from zebra fish by SDS-PAGE with alcian blue staining and confirmed that GAG’s presented a broad band of expected polydispersity. Arcanjo et al. (1994) analysed the extracellular matrix of the chicken cartilage by SDS-PAGE followed by extraction of cartilage with magnesium chloride and guanidinium chloride also observed that were more bands between 58 to 160 kDa. However, in the present study, appearance of bands between the molecular weight of 5–20 kDa, indicated that the extracted CS had the molecular weight of 5–20 kDa.

Medeiros et al. (2000) and Rocha et al. (2000) noted that extraction of CS by digestion with proteolytic enzymes was most common procedure for releasing CS from its tissue and extensive proteolysis with protease of broad specificity was desired and treatment with papain or pronase yielded single CS chains with only small residual peptides. In the present study papain used as a proteolytic enzyme to hydrolyse the cartilage for extraction of CS had shown bands in the electrophorogram which was similar to that of standard CS electrophorogram. It revealed that the molecular structure of CS had been well maintained during the extraction procedure. Aikawa et al. (1986) visualized chondroitin sulphate by isolation from porcine thoracic aorta after polyacrylamide gel electrophoresis by staining with toluidine blue. However, in the present study after electrophoresis the gel was visualised by staining with coomassie brilliant blue. Nakano et al. (2010) studied the extraction, isolation and analysis of CS glycosaminoglycan and determined CS molecular mass by polyacrylamide gel electrophoresis and also reported that the mobility of CS was depending on its charge density.

The results of current study was in accordance with the CS electrophoretic pattern identified by SDS-PAGE of duck trachea by Vittayanont and Jaroenviriyapap (2014), crocodile sternal and hyoid cartilage by Garnjanagoonchorn et al. (2007), poultry trachea by Jaroenviriyapap and Vittayanont (2009), chicken keel cartilage by Khan et al. (2013). There was no significant difference in CS electrophoresis pattern extracted from different sources. However, Nakano et al. (2010) reported that PAGE has been used to estimate molecular mass of glucosaminoglycans but, it is not much sensitive to detect it’s presence in small variation in the molecular mass, and the results of electrophoresis of CS samples had shown similar migration pattern of standard CS. This indicates that non CS proteins are effectively removed during the extraction process.

## Conclusion

The yields of CS from buffalo tracheal, nasal and joint cartilages were 62.05 ± 0.5, 60.47 ± 1.19 and 60.76 ± 0.38 mg/g of dried cartilage respectively which indicates prospective sources for utilization of these in production of chondroitin sulfate. The extraction method adopted could efficiently be utilized for the extraction of CS from buffalo by-products like tracheal, nasal and joint cartilages. FTIR spectra data has shown characteristic peaks suggestive of chondroitin sulfate similar to other common sources of production. Proper collection of buffalo by-products in an organized manner from slaughterhouses might helpful in paving way for efficient utilization of production of these type of valuable high price products.
